# Snail regulation in fibroblast-like synoviocytes by a histone deacetylase or glycogen synthase kinase inhibitor affects cell proliferation and gene expression

**DOI:** 10.1371/journal.pone.0257839

**Published:** 2021-09-28

**Authors:** Po-Chuan Shen, Po-Chun Chang, Jeng-Long Hsieh

**Affiliations:** 1 Department of Orthopedics, Tainan Hospital, Ministry of Health and Welfare, Tainan, Taiwan; 2 Department of Nursing, College of Nursing, Chung Hwa University of Medical Technology, Tainan, Taiwan; 3 Department of Orthopedics, An Nan Hospital, China Medical University, Tainan, Taiwan; Chang Gung University, TAIWAN

## Abstract

**Background:**

Snail has been linked to the pathogenesis of rheumatoid arthritis (RA). We plan to investigate the regulation of Snail in response to TNF-α, histone acetylation, and glycogen synthase kinase-3 (GSK)-3 inhibition in fibroblast-like synoviocytes (FLSs).

**Methods:**

FLSs from rats with collagen-induced arthritis (CIA) were collected and treated with TNF-α alone or a combination with trichostatin A (TSA), a pan-histone deacetylase inhibitor and lithium chloride (LiCl), a glycogen synthase kinase-3 (GSK)-3 inhibitor.

**Results:**

We demonstrated for the first time that nuclear expression of Snail in FLSs from rats with CIA was correlated with the levels of extracellular TNF-α and acetylation status. Cell proliferation and viability of CIA FLSs were reduced in response to TSA treatment and short-hairpin RNA specific to Snail. LiCl treatment increased Snail and cadherin-11 (Cad-11) expression in CIA FLSs.

**Conclusion:**

We suggested from this study that targeting TNF-α-histone deacetylase-Snail signaling axis or the Wnt signaling pathway in FLSs might provide therapeutic interventions for the treatment of RA in the future.

## Introduction

In rheumatoid arthritis (RA), fibroblast-like synoviocytes (FLSs) behave like transformed cells, as demonstrated by growing *in vitro* in an anchorage-independent manner [1] and maintaining their invasive and destructive behaviors *in vivo* [2], which are responsible for destructive arthritis [3]. Epithelial-mesenchymal transition (EMT), a phenomenon used to describe cancer cell migration and invasion, might be implicated in the invasive phenotype of RA FLS, which remained largely unknown and required further investigations [4]. Snail, a zinc finger transcription factor family member and an inducer of EMT in a variety of disease models [5,6], has been demonstrated to play crucial roles in mediating joint destruction and the pathogenesis of RA [7,8]. Chen and colleagues revealed that Snail regulated activation of FLS by lentivirus-based overexpression and knockdown strategies [7]. The proinflammatory cytokine TNF-αenhances activation of the Wnt signaling pathway by inhibiting glycogen synthase kinase-3 (GSK)-3 activation [7]. The Wnt signaling can further inactivate GSK-3-mediated phosporylation of Snail with enhancement of its stabilization [9,10]. TNF-α can also stabilize Snail by promoting its acetylation [11]. Furthermore, etanercept, a well-known TNF-α blockade, abrogated acetylated Snail-induced target gene expression [11]. Etanercept also reduces the expression of Snail in the joints of rats with collagen-induced arthritis (CIA) [7]. Taken together, these studies emphasized a critical role of TNF-α by which it might serve as an extracellular stimulator to induce the stabilization of Snail.

Histone deacetylase (HDAC) has long been linked to the pathogenesis of RA. Increased expression and activity of HDAC1 have been identified in synovium of patients with RA and related to local TNF-α levels [12]. TNF-α induces HDAC1 mRNA and protein expression as well as enzyme activity in RA FLSs [12]. These results implicated the cytokine-induced HDAC1 might participate in RA pathogenesis via mediating acetylation of histone and non-histone substrates. Interestingly, HDAC inhibitor can suppress autoantibody-mediated arthritis in mice through induction of p16 or p21 that controls cell-cycle and reduces cell proliferation of RA FLSs [13]. Furthermore, trichostatin A(TSA), a commonly used HDAC inhibitor that can restore histone acetylation and contribute to gene expression [14], has been shown to sensitize RA FLSs for TRAIL-induced apoptosis by inducing p21 expression [15]. Silencing of HDAC1 resulted in reduced cell proliferation, invasion and migration of FLSs as well as amelioration of CIA [15,16]. Unifying these observations, we propose that deacetylation of certain therapeutic proteins underling the repression of HDAC may have pathological effects on chronic arthritis. However, treatment of the HDAC inhibitor can increase the acetylation status and protein stability in conjunction with suppression of arthritis.

To further address the concepts, fresh FLSs were isolated from synovium of rats with CIA, and were further treated with trichostatin A(TSA), a well-known pan-histone deacetylase inhibitor, and lithium chloride (LiCl), a GSK-3 inhibitor, to observe their Snail expression levels and cell responses.

## Materials and methods

### Induction of CIA and isolation of FLSs

The animal experiment was done strictly in accordance with protocols approved by the Institutional Animal Care and Use Committee of National Cheng Kung University (No. 108059). CIA was induced in Male Sprague-Dawley rats (~8 weeks of age), which were immunized with bovine type II collagen and Freund’s complete adjuvant, as described previously [7]. After sacrificed with overdose of CO_2_ to minimize suffering, fresh FLSs were isolated from rat synovium with CIA, and different lines between the fourth and seventh passages were used [7].

### Immunoblot and immunofluorescence analyses

CIA FLSs were treated simultaneously with TSA (2 μM, Sigma-Aldrich) and TNF-α (10 ng/ml, PeproTech) for 24h, and dimethyl sulfoxide(DMSO) and LiCl (Merck), and their cell lysates were subjected to immunoblot analysis with anantibody against Snail (Cell Signaling Technology) in combination with an horseradish peroxidase–conjugated secondary antibody (JacksonImmunoResearch) and quantitative control anti-β-actin antibodies (Sigma-Aldrich). Immunofluorescence assessment was performed with the Snail antibody as described above, followed by a fluorescein isothiocyanate–conjugated secondary antibody (SeraCare KPL).

### Preparation of lentiviral vectors and stable CIA FLS transfactants in which Snail is silenced

Lentiviral vectors expressing short-hairpin RNA (shRNA) specific to either Snail (shSnail#218784, shSnail#234035, and shSnail#96619) or luciferase were produced by transient transfection of pLKO.1-shSnail (TRCN0000218784, TRCN0000234035, and TRCN000096619) and luciferase shRNA–expressingpLKO.1-shLuc (TRCN0000072246) lentiviral lasmids, along with the packaging plasmid psPAX2 and the envelope plasmid pMD2G into 293T cells, as previously published by Chen et al [7]. To generate stable transfectants in which Snail is silenced, CIA FLSs were transduced with lentiviral vector expressing shSnail (LVshSnail) or shluciferase (LVshLuc), and selected with 2μg/ml of puromycin for almost 2 weeks, as described by Chen et al [7].

### Real-time PCR analysis

Total RNA from LVshSnail and LVshLuc-transduced CIA FLSs was isolated with TRIzol reagents (Invitrogen), and complementary DNA was synthesized with a Reverse-iT First Strand cDNA synthesis kit (ABgene) for real-time PCR by SYBRGreen PCR kit(Qiagen) with primer pairs specific to Snail(forward, 5’-CCGGAAGCCCAACTATAGCG-3’, and reverse 5’-AAGGTGAACTCCACACACGC-3’, and GAPDH(forward 5’- GACTCTACCCACGGCAAGTT-3’, and reverse 5’-GGTGATGGGTTTCCCGTTGA-3’), respectively. The comparative Ct method was used to calculatethe relative abundance of Snail gene compared with GAPDH expression.

### Cell proliferation and viability analyses and enzyme-linked immunosorbent assay (ELISA)

CIA FLSs were treated with a serial dose of TSA (0.2, 1, and 2 μM) and DMSO as the control group. Cell proliferation was determined by WST-8 analysis 24 and 48h after TSA treatments. For viability assay, stable CIA FLS transfectants transduced with LVshSnail or LVshLuc were subjected to WST-8 analysis. Supernatant from LVshSnail or LVshLuc -transduced stable CIA FLS transfectants were subjected to ELISA (R&D Systems) for detecting VEGF expression levels.

### Statistical analysis

Data were expressed as the mean ±SEM. Differences among groups were analyzed using one-way ANOVA followed by Dunnet multiple comparison tests. *P*<0.05 was considered statistically significant.

## Results

### TSA decreases Snail expression and cell proliferation in TNF-α-stimulated CIA FLSs

**Fig 1:**
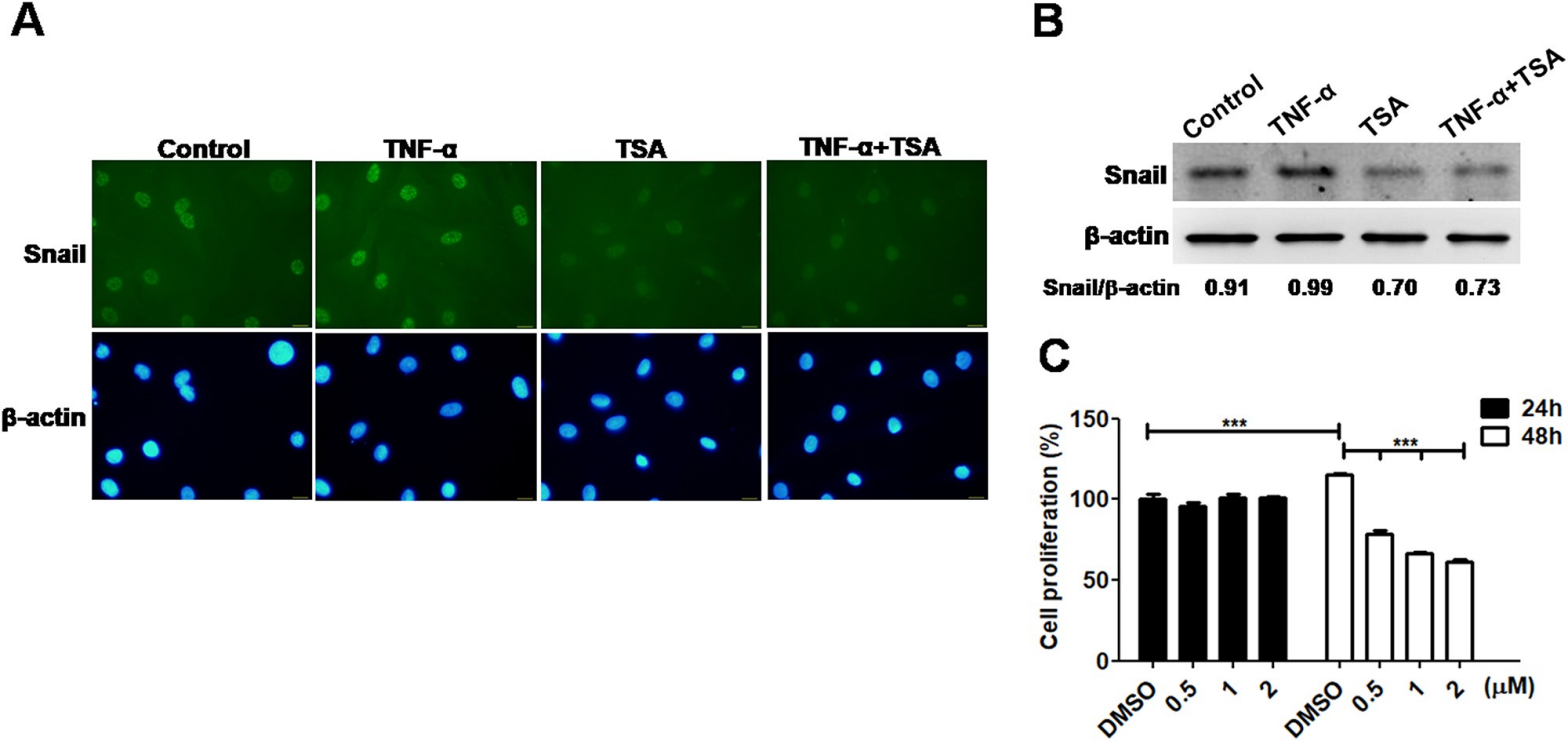
Expression of Snail and cell proliferation in fibroblast-like synoviocytes (FLSs) from rats with collagen-induced arthritis (CIA) in response to trichostatin A (TSA) (2 μM) and TNF-α (10 ng/ml) stimulation for 24h, as observed by (A) fluorescence microscope and (B) immunoblot analysis. Control cells were treated with dimethyl sulfoxide (DMSO). DAPI represents nuclear staining (C) cell proliferation was determined at different doses (0.5–2 μM of TSA) and time points (24 and 48h) by WST-8 analysis. Values were expressed as mean ± SEM.***p<0.001. Scale bars represent 50 μm in × 400 magnifications.

Interestingly, the nuclear expression of Snail in TSA-treated CIA FLSs was decreased in response to TNF-α stimulation, as determined by immunofluorescent staining (Fig 1A) and immunoblotting (Fig 1B). Fig 1C showed cell proliferation was reduced in TSA-treated CIA FLSs dose-dependently (0.5–2 μM of TSA) at 48h when compared to DMSO-treated control cells, as determined by WST-8 analysis.

### Regulation of Snail influences cell viability, vascular endothelial growth factor (VEGF), and cadherin-11 (Cad-11) expression in CIA FLSs

**Fig 2:**
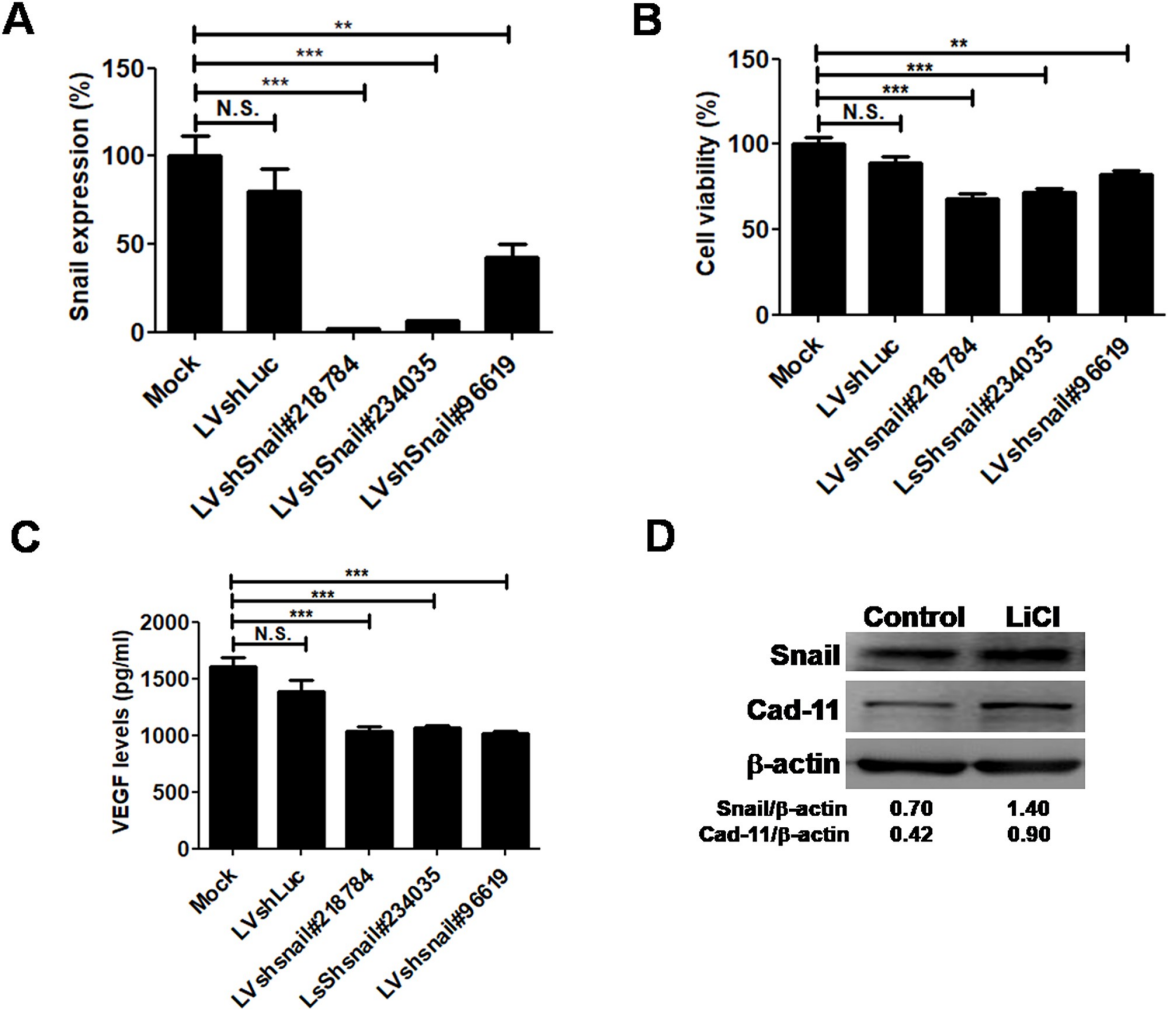
Snail, vascular endothelial growth factor (VEGF), and cadherin-11 (Cad-11) expressions and cell viability in short-hairpin RNA (shRNA) and lithium chloride(LiCl)-treated CIA FLSs. (A) Real-time PCR for Snail in FLSs from CIA rats transduced with lentiviral vectors expressing Snail(LVshSnail) and luciferase (LVshLuc)-specific shRNA. (B) Cell viability was determined by WST-8 analysis. (C) VEGF expression levels in LVshLuc and LVshSnail-transduced CIA FLSs. (D) Snail, Cad-11, and β-actin expressions in LiCl (10 mM) and DMSO-treated CIA FLSs for 24h. Values were expressed as mean ± SEM. N.S., not significant, **p<0.01, ***p<0.001.

To determine if reduction of Snail expression could result in down-regulation of gene expression and decrease of cell viability, we infected CIA FLSs with lentiviral vectors expressing shRNA against Snail and luciferase, designated LVshSnail and LVshLuc, as published by Chen and colleagues (1). LVshSnail-transduced FLSs have silenced Snail expression, lower cell viability and vascular endothelial growth factor (VEGF) expression than LVSin-treated control cells (Fig 2A–2C). However, LiCl-treated FLSs have higher Snail and Cad-11 expression than control-treated counterparts, indicating that GSK-3 activity was involved in regulating both Snail and Cad-11 expression (Fig 2D).

## Discussion

Our results raise the possibility that theTNF-α-HDAC-Snail signaling axis may play pathogenic roles in RA through deacetylation of Snail and contribution to activation and cell proliferation of FLSs (Fig 1). Furthermore, Snail-silenced CIA FLSs have reduced levels of VEGF and cell viability (Fig 2B and 2C) and GSK-3 activity also contributes to regulation of Snail (Fig 2D). These findings may provide the development of pharmacologic therapies targeting Snail and its complex regulatory network, including TNF-α-HDAC-Snail axis and the Wnt signaling pathway in FLSs from patients with RA.

In the rheumatoid joint, monocytes/macrophages are the source of TNF-α or IL-1 that trigger activation of resident FLS to release growth factors, such as granulocyte-macrophage colony stimulating factor and colony stimulating factor-1 and IL-6, which in turn amplify the inflammatory loop by inducing TNF-α release in macrophages [17,18]. TNF-α has been proven to activate the Wnt pathway by evaluating phospho-GSK-3 expression in CIA FLSs [7]. Furthermore, treatment of the specific GSK-3 inhibitor (SB216763) in FLSs induces the expression of both Snail and Cad-11 [7], as observed by LiCl-treated FLS in our study (Fig 2D). Zhou and colleagues also used LiCl as an activator of the Wnt/β-catenin signaling pathway to study TNF-α-stimulated RA FLS (MH7A cells) proliferation, migration, and invasion [19]. This indicated that TNF-α is crucial for Snail induction via the Wnt pathway through stabilization of Snail [7]. In the present study, we provided further information about histone acetylation that mediated the TNF-α-induced Snail expression in CIA FLSs and proposed that theTNF-α-HDAC-Snail axis was involved in FLS proliferation, which had not been confirmed before.

Cad-11 is essential for the development of synovium and can synergize with TNF-α to promote IL-6 production in FLSs [20,21]. Furthermore, IL-1β-induced FLS proliferation can be through the Cad-11-mediated β-catenin signaling [22], which is a crucial adaptor of the Wnt pathway [23] Besides, Snail may directly regulate the transcriptional activation of Cad-11 gene, in which the promoter region contains three zinc-finger binding motifs that are presumably regarded as binding sequences for Snail, as predicted by the PROMO software [7,24]. We suggested this axis might also promote FLS proliferation through transcriptional activation of Cad-11 by Snail, which required further investigations.

Taken together, we synthesized a conclusion that TNF-α-HDAC-Snail axis and the Wnt/β-catenin signaling pathway were involved in regulation of Snail and cell proliferation of arthritic FLS. The crosstalk between the two pathways would worthily be addressed, which might contain critical issues and help to design effective therapeutic intervention for RA.

## Supporting information

S1 Raw images Raw images of immunoblot analyses. (PDF)

S1 File The values used to build graphs. (PDF)
