# Supplementary figures and images for: Snail regulation in fibroblast-like synoviocytes by a histone deacetylase or glycogen synthase kinase inhibitor affects cell proliferation and gene expression
Source: PLoS One. 2021 Sep 28;16(9):e0257839. doi: 10.1371/journal.pone.0257839 (PMC8478242; doi:10.1371/journal.pone.0257839)

Figure 1B

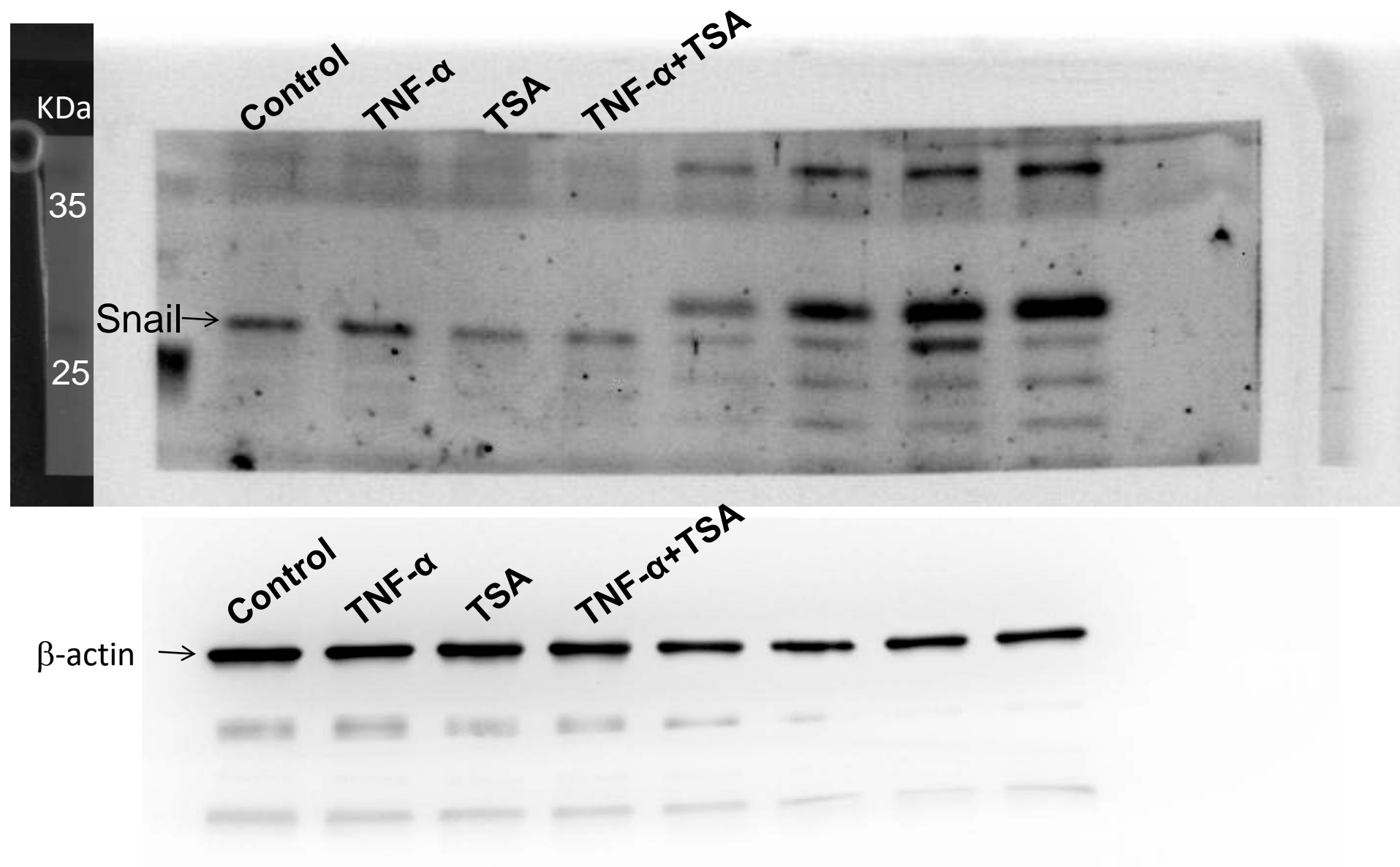

Figure 2D

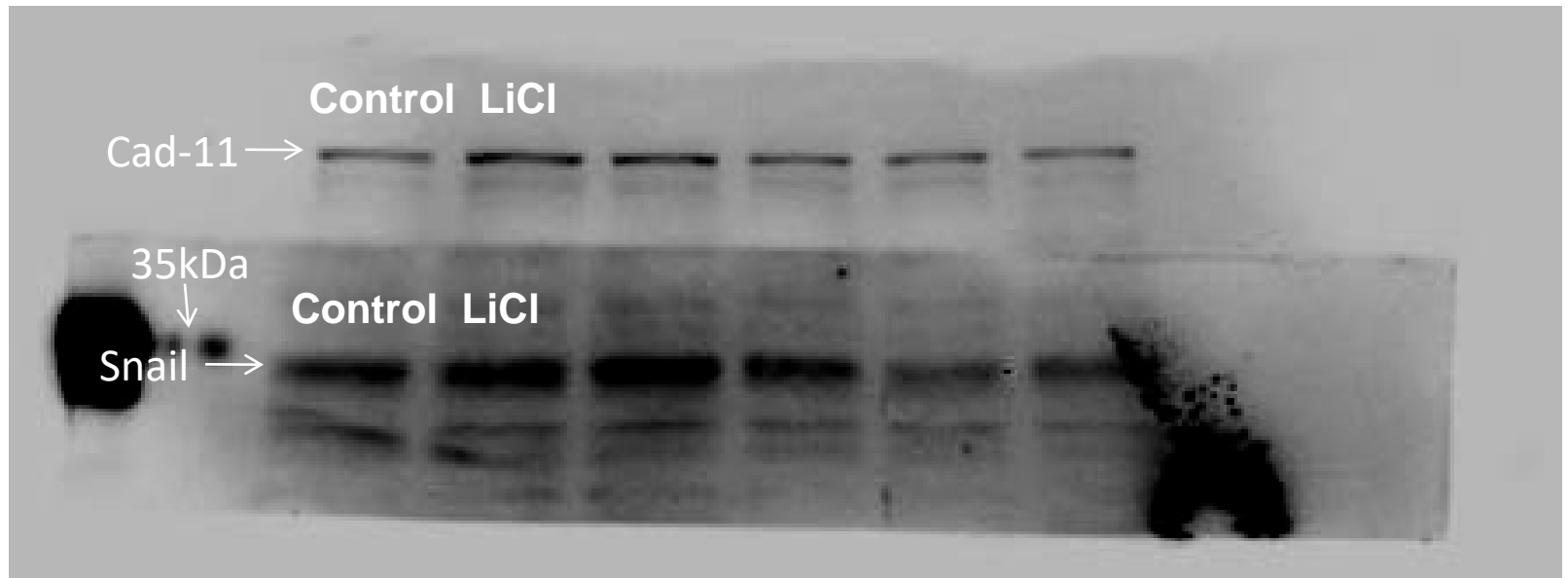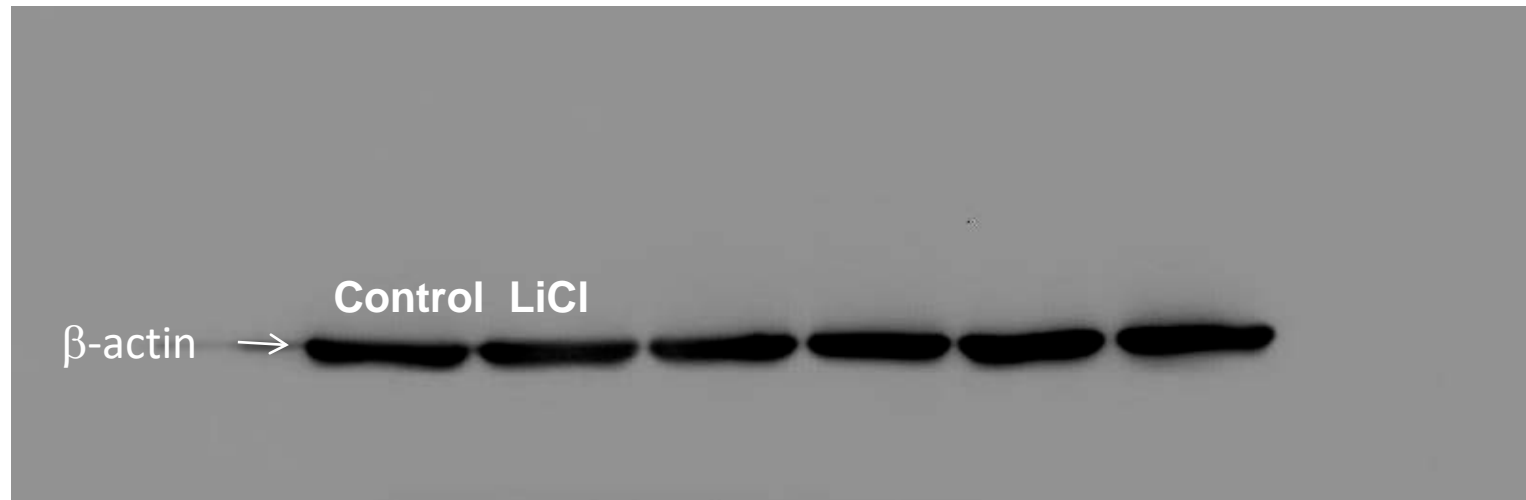

Supplement: S1 Raw images — (PDF) [file pone.0257839.s001.pdf]
