# Supplementary material for: Snail regulation in fibroblast-like synoviocytes by a histone deacetylase or glycogen synthase kinase inhibitor affects cell proliferation and gene expression
Source: PLoS One. 2021 Sep 28;16(9):e0257839. doi: 10.1371/journal.pone.0257839 (PMC8478242; doi:10.1371/journal.pone.0257839)
Supplement: S1 File — (PDF) [file pone.0257839.s002.pdf]

Table format: Grouped	A 24h				B 48h			
	A:Y1	A:Y2	A:Y3	A:Y4	B:Y1	B:Y2	B:Y3	B:Y4
1 DMSO		94.987780	99.022000	105.990200	114.425400		114.425400	116.259200
2 0.5	94.621030	92.787290	100.122200	96.821520	75.183370		82.518340	78.117360
3 1	105.990200	96.454770	102.322700	98.288510	64.914430	66.014670	68.948650	66.381420
4 2	102.322700	101.956000	100.855700	99.755500	60.146700	65.281170	61.613690	57.579460

Fig 1C cell proliferation was determined at different doses (0.5-2 μM of TSA) and time points (24 and 48h) by WST-8 analysis. Values were expressed as mean ± SEM

	A Mock	B LVshLuc	C LVshSnail#218784	D LVshSnail#234035	E LVshSnail#96619
	Y	Y	Y	Y	Y
1	79.806670	58.421900	1.472673	6.490985	48.450430
2	118.474900	101.015800	2.171117	6.627375	28.808800
3	101.718400	81.483580	1.890067	6.581596	51.212970

Fig 2A Real-time PCR for Snail in FLSs from CIA rats transduced with lentiviral vectors expressing Snail (LVshSnail) and luciferase (LVshLuc)-specific shRNA Values were expressed as mean ± SEM

	A	B	C	D	E
	Mock	LVshLuc	LVshsnail#218784	LsShsnail#234035	LVshsnail#96619
	Y	Y	Y	Y	Y
1	97.9372	91.2407	69.5964	77.0105	83.7070
2	107.8625	79.0433	72.1076	71.5097	74.3797
3	90.4036	91.1211	60.3886	63.3782	83.3483
4	103.7967	94.8281	70.7922	73.7818	87.0553
5					

Fig 2B Cell viability was determined by WST-8 analysis. Values were expressed as mean ± SEM

	A	B	C	D	E
	Mock	LVshLuc	LVshsnail#218784	LsShsnail#234035	LVshsnail#96619
	Y	Y	Y	Y	Y
1	1734.8100	1186.4400	996.3360	1059.1400	985.5710
2	1695.5000	1320.3400	1105.4300	1095.6500	1066.7400
3	1376.8400	1663.0000	1103.4600	1103.4600	1019.9800
4	1616.9300	1357.7500	948.6110	999.9450	1009.0100
5					

Fig 2C VEGF expression levels in LVshLuc and LVshSnail-transduced CIA FLSs Values were expressed as mean ± SEM
